# Histological characterization of orphan transporter MCT14 (SLC16A14) shows abundant expression in mouse CNS and kidney

**DOI:** 10.1186/s12868-016-0274-7

**Published:** 2016-07-01

**Authors:** Sahar Roshanbin, Frida A. Lindberg, Emilia Lekholm, Mikaela M. Eriksson, Emelie Perland, Johan Åhlund, Amanda Raine, Robert Fredriksson

**Affiliations:** Department of Neuroscience, Functional Pharmacology, Uppsala University, 75124 Uppsala, Sweden; Department of Pharmaceutical Biosciences, Molecular Neuropharmacology, Uppsala University, 75124 Uppsala, Sweden

**Keywords:** Monocarboxylate, Transporter, Mouse, Amino acid

## Abstract

**Background:**

MCT14 (SLC16A14) is an orphan member of the monocarboxylate transporter (MCT) family, also known as the SLC16 family of secondary active transmembrane transporters. Available expression data for this transporter is limited, and in this paper we aim to characterize MCT14 with respect to tissue distribution and cellular localization in mouse brain.

**Results:**

Using qPCR, we found that *Slc16a14* mRNA was highly abundant in mouse kidney and moderately in central nervous system, testis, uterus and liver. Using immunohistochemistry and in situ hybridization, we determined that MCT14 was highly expressed in excitatory and inhibitory neurons as well as epithelial cells in the mouse brain. The expression was exclusively localized to the soma of neurons. Furthermore, we showed with our phylogenetic analysis that MCT14 most closely relate to the aromatic amino acid- and thyroid-hormone transporters MCT8 (SLC16A2) and MCT10 (SLC16A10), in addition to the carnitine transporter MCT9 (SLC16A9).

**Conclusions:**

We provide here the first histological mapping of MCT14 in the brain and our data are consistent with the hypothesis that MCT14 is a neuronal aromatic-amino-acid transporter.

**Electronic supplementary material:**

The online version of this article (doi:10.1186/s12868-016-0274-7) contains supplementary material, which is available to authorized users.

## Background

The solute carrier (SLC) superfamily is currently comprised of 456 members divided into 52 families [1], making it the largest group of transporters. The common features of transporters in this superfamily are their secondary active mode of transport across membranes and their wide substrate profile, making them key in many biological processes, diseases and therapies [2]. Members are divided into SLC families on the basis of at least 20 % sequence similarity [3, 4]. The SLCs can be further divided into four phylogenetic clusters on the basis of their evolutionary origin; the largest of these clusters is the α-group containing eight SLC-families [5]. The SLC16 gene family is part of the α-cluster and is currently comprised of 14 members, all predicted to have 12 transmembrane helices (TM) with intracellular C- and N-termini, and a large cytosolic loop between TMs 6 and 7 [6–8]. The substrates for the first characterized members of this family of transporters are amino acids, carbohydrates, and monocarboxylates [9]. The SLC16 protein family is therefore also called the monocarboxylate transporters (MCTs). The SLC36 [10] and SLC5 [11, 12] are other SLC-families involved in the transport of monocarboxylates. Only six of the fourteen members of the MCT family have been characterized in detail: SLC16A1, SLC16A7, SLC16A8, SLC16A13, SLC16A2 and SLC16A10, corresponding to MCT1–4, MCT8 and MCT10 respectively. The inconsistent nomenclature is due to the naming of MCTs in the order in which they were functionally characterized, whereas SLC16-family members were named as their cDNA sequences became available [6].

The members of the MCT family share structural similarities but differ in substrate specificity. MCTs 1–4 [13, 14] are the most extensively characterized, and display a broad specificity for monocarboxylates such as pyruvate, ketone bodies, lactate, and short-chain fatty acids. The transport facilitated by these transporters is proton-linked. MCT8 transports thyroid hormones, MCT10 transports thyroid hormones and aromatic amino acids, and both display a proton-independent mode of transport [15–17], further supported by their lack of the proposed proton-binding domain [13, 14] present in MCT1–4. Interestingly, MCT6 also lacks this domain, but it facilitates the proton-linked transport of the synthetic drug bumetanide as well as probenecid. However, the transport mechanism and the endogenous substrates for MCT6 are still unknown [18]. MCT9 has been identified as a carnitine transporter [19, 20] with an unknown mode of transport. As for MCT14, its substrate profile is still unknown and the expression data are limited. MCT14 has been found in bovine mammary gland, in the gastrointestinal tracts of cattle and the mammary glands of lactating cows [21–23], but there is no detailed immunohistochemical analysis of MCT14 expression in mouse brain.

In this paper we show that *Slc16a14* is phylogenetically most closely related to *Slc16a9*, *Slc16a2* and *Slc16a10*. We also mapped the expression of MCT14 throughout the mouse brain on an mRNA level using an antisense RNA probe. We determined the regional protein distribution using a verified MCT14 antibody and established that MCT14 expression is confined to soma of mainly excitatory, but also inhibitory neurons, as well as epithelial cells. Quantitative real-time PCR (qRT-PCR) was used to analyze the relative expression levels of *Slc16a14* mRNA in a panel of mouse tissues and defined brain regions, where we found high expression of *Slc16a14* in the kidney and moderate levels in the brain, testis, uterus and liver.

## Methods

### Phylogenetic analysis

**Fig. 1:**
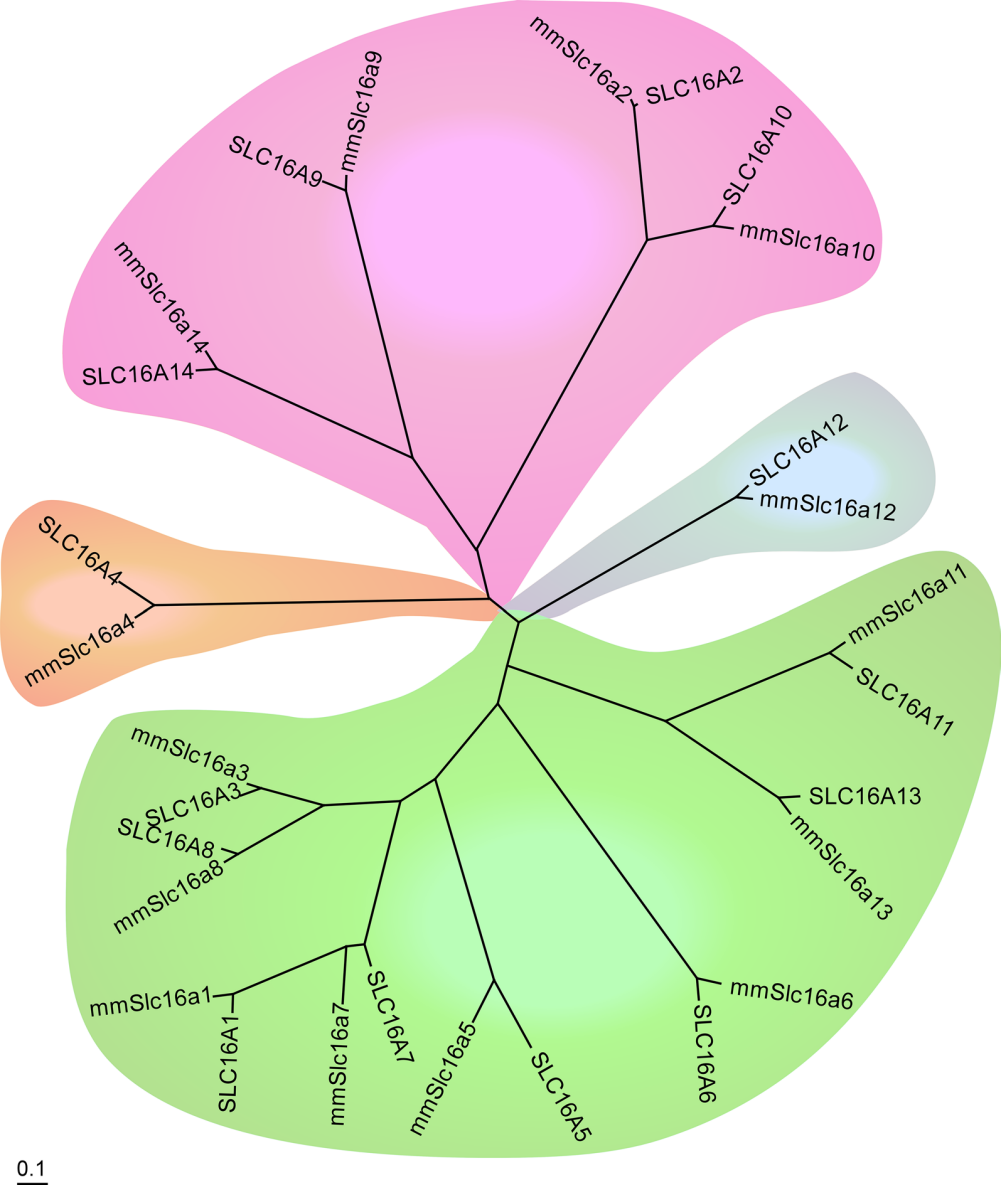
*Slc16a14* is evolutionarily most closely related to *Slc16a9*, *Slc16a2* and *Slc16a10*. Phylogenetic tree showing the evolutionary relationship between the members in the mouse and human monocarboxylate transporter family. MrBayes 3.2.2 [25, 26] was used to construct the tree with branch lengths representing evolutionary distance. The *scale bar* represents 0.1 exchanges per site

All human and mouse SLC16 amino acid sequences were identified [14] and combined into a multiple sequence alignment using t_coffee [24]. The phylogenetic relationships between these sequences were inferred using the Bayesian approach as implemented in mrBayes 3.2.2 [25, 26] to obtain the tree in Fig. 1. The analysis was run via the Beagle library [27] on 2× AMD 290× graphics cards. The analysis was run on six chains (five heated and one cold) with two runs in parallel (n runs = 2) under the mixed amino acid model with eight gamma categories and invgamma as gamma rates for a total of 2,000,000 generations. A maximum likelihood analysis was also performed using RAxML [28] with 1000 bootstrap replicas and the GAMMAJTT protein model. The best tree from the maximum likelihood analysis had the same topology as the one obtained from MrBayes.

### Tissue preparation

Tissue preparation for in situ hybridization (ISH) and immunohistochemistry (IHC) was performed as previously described [29].

### RNA-probe synthesis and in situ hybridization

Mouse *Slc16a14* EST clone ID IRAVp968E0963D was used to synthesize the antisense *Slc16a14* probe, of which 1 µg/ml was used in the ISH, described in [29].

### Immunhisto- and cyto-chemistry

IHC with anti-MCT14 on free floating sections (1:1000, non-fluorescent IHC) and paraffin sections (1:100, fluorescent IHC) was performed as previously described in [29], using primary polyclonal rabbit anti-MCT14 (Sigma-Aldrich Cat# HPA040518, RRID:AB_10794877). For the peptide-blocked control section, MCT14 antibody (1:100) was pre-incubated with a peptide that corresponds to the epitope recognized by the antibody (YTSHEDIGYDFEDGPKDKKTLKPHPNIDGG, Sigma-Aldrich, cat. no. APREST79171) in excess (5:1 to antibody concentration,) for 1 h at room temperature prior to incubation. The immunocytochemistry (ICC) also followed the procedures described in [29], with the exception of the cell line used; here fixed wild-type PC12 cells were used, and transfected PC12 cells were stained with anti-MCT14 and primary monoclonal mouse anti-FLAG M2 antibody (1:200, Sigma-Aldrich, cat. no. F3165, RRID:AB_259529).

### Colocalization analysis

Double IHC of MCT14 with NeuN and GAD67 was performed as described previously [29]. MCT14/NeuN and MCT14/GAD67 images of hypothalamus, retrosplenial cortex and piriform cortex from a total of six sections for each region (n = 2) were acquired with a Zeiss AxioImager widefield fluorescence microscope at 20× magnification. All images were analyzed using a specialized pipeline in the automated open-source cell segmentation software CellProfiler (Additional file 1: Figure S1) (Broad Institute Imaging Platform, Cambridge, MA, USA) [30]. For an overview of the analysis pipeline and details regarding incorporated modules, see Additional file 2.

### Western Blot

Verification of the MCT14 antibody through Western Blot was performed according to procedures described in [29], with the exception of the antibody, the gel, and the blotting procedure. Proteins were resolved on Mini-Protean 4–15 % TGX Stain-Free^®^ gels and transferred to polyvinylidene difluoride membranes using Trans-Blot^®^ Turbo™ Mini PVDF Transfer Packs and Trans-blot Turbo Transfer system (Bio-Rad Laboratories, Sweden) and incubated with anti-MCT14 (1:100). For peptide-blocking, anti-MCT14 (1:300) was pre-incubated with the corresponding peptide according to procedures described for IHC. Concentration of mouse brain protein sample was 20 µg, and to ensure equal loading of protein, β-actin (1:50,000, Sigma-Aldrich, Cat# A1978, RRID:AB_476692) was used. Band intensities were quantified by densitometry using ImageLab™ software (version 4.1 BioRad Hercules, CA, USA).

### Transfection of PC12 cells

The immortalized rat adrenal gland cell line PC-12 Adh (ATCC, Manassas, VA, USA, CRL-1721.1) was cultured in ATCC-formulated complete growth media F-12 K (ATCC, Manassas, VA, USA cat. no. 30-2004) supplemented with 12.5 ml fetal bovine serum (FBS), 75 ml horse serum, 5 ml penicillin–streptomycin (Pen-Strep) and 5 ml amphotericin B (all from Gibco, Life Technologies, Sweden). All cells were incubated at 37 °C with 5 % CO_2_. The cells were seeded to 60–70 % confluence on cover slips coated with 10 μg/ml poly-l-lysine (Sigma-Aldrich, cat. no. P4707). Cells were transfected with one µg of a pcDNA3.1 + DYK vector containing the full *Slc16a14* mRNA sequence, as well as a FLAG tag sequence (Additional file 3: Figure S2, DYKDDDK, Clone ID: OMu04791, Genscript Piscataway, NJ, USA), using Lipofectamine Plus reagent (Invitrogen, Sweden), according to the manufacturer’s instructions. Cells were fixed in 4 % formaldehyde 48 h post-transfection.

### RNA preparation and cDNA synthesis

Central- and peripheral-tissues were isolated from wild-type male mice (n = 4) while females (n = 4) were euthanized for female genitalia in order to create a wild-type tissue panel. Mice were killed by cervical dislocation, and dissections were performed on ice within 10 min. Tissues were kept in RNA-later (Qiagen, Denmark) for 2 h at room temperature, before storage at −80 °C. Blood was collected via a cardiac puncture after euthanizing the animal. The blood was mixed with EDTA (1.5 mg/ml blood, VWR, Sweden), centrifuged for 10 min (4 °C, 21,100×*g*) and the pellet was used for RNA extraction. RNA Extraction Absolutely RNA Miniprep Kit (Agilent Technologies, USA) was used to extract RNA from individual samples. Briefly, tissues were homogenized in lysis buffer and β-mercaptoethanol using a Bullet blender (Next Advance Inc., NY, USA). The homogenate was run through a prefilter spin cup to remove non-homogenized tissue before precipitation of the RNA with 70 % ethanol (Solveco, Sweden). The RNA was collected in an RNA-binding spin cup and washed in salt buffers before incubation in DNase Digestion buffer and RNaseFree DNase 1 for 15 min at 37 °C. Additional salt buffer washes were performed prior to RNA elution. Concentration was measured in an ND-1000 spectrophotometer (NanoDrop Technologies Inc., Wilmington, DE, USA). One µg extracted RNA was used as template for the cDNA synthesis. The High Capacity RNA-to-cDNA kit (Invitrogen, Sweden) was used in which the RNA was added to 2RT Reaction mix and RT enzyme mix. The final volume was adjusted to 20 µl with DEPC-treated water before the cDNA synthesis cycle: 10 min incubation at 25 °C, 30 min at 50 °C and 85 °C for 5 min. Samples were cooled before two units *Escherichia coli* RNase H was added, followed by an additional 20 min incubation at 37 °C. cDNA from the same organs of different animals, were pooled and diluted to 5 ng/µl RNA with sterile water.

### Primer design and quantitative real-time PCR (qRT-PCR)

All primers were designed using Beacon Design 8 (Premier Biosoft, Palo Alto, CA, USA). For sample amplification the primers used were Slc16a14 forward 5′-tgaagacgaccgaaaggctaa-3′ and reverse 5′-atgtgaacaaagaaggacgagag-3′. Six different reference genes were run: glyceraldehyde-3-phosphate dehydrogenase (Gapdh) forward 5′-gccttccgtgttcctacc-3′, reverse 5′-gcctgcttcaccaccttc-3′, beta tubulin 4B (bTub) forward 5′-agtgctcctcttctacag-3′, reverse 5′-tatctccgtggtaagtgc-3′, ribosomal protein L19 (Rpl19) forward 5′-aatcgccaatgccaactc-3′, reverse 5′-ggaatggacagtcacagg-3′, pepti-dylprolyl isomerase A (Cyclo) forward 5′-tttgggaaggtgaaagaagg-3′, reverse 5′-acagaaggaatggtttgatgg-3′ and actin-related protein 1B (Actb) forward 5′-ccttcttgggtatggaatcctgtg-3′, reverse 5′-cagcactgtgttggcatagagg-3′. Final volume for each qRT-PCR reaction was 20 μl; 1 μl pooled cDNA (5 ng/μl RNA), 0.05 μl of each primer (100 pmol/μl), 3.6 µl 10× DreamTaq buffer (including 20 mM MgCl_2_; Thermo Scientific), 0.2 μl of 25 mM dNTP mix (Thermo Scientific, Sweden), 1 μl DMSO, 0.5 μl of SYBR Green (1:10,000; Invitrogen, Sweden) diluted in TE buffer (pH 7.8) and 0.08 μl of DreamTaq DNA polymerase (5 U/µl, Thermo Scientific, Sweden). The iCycler real-time detection instrument (Bio-Rad Laboratories, Sweden) was used and the reaction followed these conditions: initial denaturation for 3 min at 95 °C followed by 45 cycles of 10 s at 95 °C, 30 s at 55 or 60 °C (55 °C for housekeeping genes; 60 °C for Slc16a14:) and 30 s at 72 °C, followed by further elongation at 72 °C for 5 min. Thereafter a melting curve was performed by 81 cycles of 10 s intervals where the temperature increased 0.5 °C per cycle, starting from 55 °C. All RT-PCR reactions were performed in triplicate; in addition, a negative control was included on each plate. All experiments were repeated twice.

### qRT-PCR data analysis

All data were collected using MyIQ (Bio-Rad Laboratories, Hercules, CA, USA) software. Primer efficiency was calculated for each run using LinRegPCR software, followed by the Grubbs test (GraphPad software, version 5.0, La Jolla, CA, USA) to remove outliers in the efficiency calculations before correcting the samples for primer efficiency. The GeNorm protocol [31] identified five stable housekeeping genes, and their geometric means were used as normalization factors for each tissue. Normalized mRNA expression (±SD) was plotted.

## Results

### Slc16a14 is evolutionarily most closely related to Slc16a9, Slc16a2 and Slc16a10

Phylogenetic reconstruction of the evolutionary relationship of all mouse and human SLC16 sequences (Fig. 1) showed that *Slc16a14* is most closely related to *Slc16a9*, a carnitine transporter [19, 20]. The *Slc16a9* and *Slc16a14* group form a sister clade to the *Slc16a2* and *Slc16a10* group, which are transporters for thyroid hormone (*Slc16a2*) [16, 32] and aromatic amino acids as well as iodothyronine (*Slc16a10*) [33, 34]. Identical topology was obtained with maximum likelihood analysis using RAxML [35] (data not shown).

### Extensive Slc16a14 mRNA expression in mouse brain

**Fig. 2:**
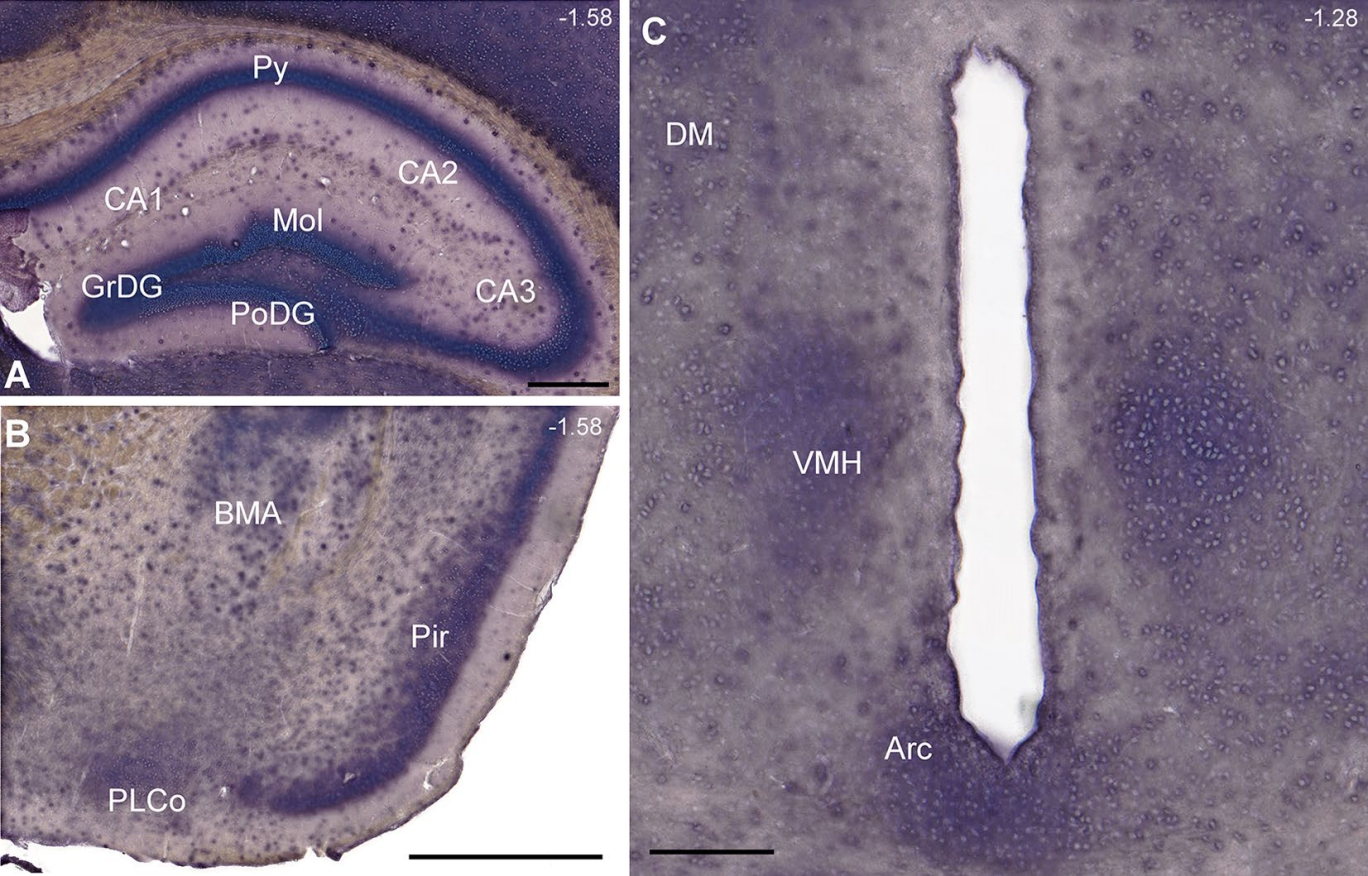
Extensive *Slc16a14* mRNA expression in mouse brain. ISH with a digoxigenin-labeled riboprobe against *Slc16a14* mRNA was used to map the expression in mouse brain through hybridization with free-floating, 70-µm coronal sections. Widespread expression was seen in various parts of the mouse brain, including the: **A** granular, molecular and polymorph layers of the dentate gyrus (GrDG, Mol and PoDG respectively), CA1–3 fields and pyramidal cells of the hippocampus, **B** piriform cortex (Pir), accessory basal amygdaloid nucleus (BMA) and posterolateral cortical amygdaloid nucleus, **C** arcuate nucleus (Arc) and ventromedial and dorsomedial hypothalamic nuclei (VMH and DM respectively). All *scale bars* represent 200 µm. Bregma coordinates (*upper left corner* of each image) and abbreviations were determined according to Franklin and Paxinos [57]

ISH with a digoxigenin-labeled riboprobe against *Slc16a14* mRNA was used to map the expression in mouse brain through hybridization with free-floating 70 µm coronal sections (Fig. 2). Abundant mRNA was detected throughout the mouse brain, exemplified in the close-up images in Fig. 2. Widespread expression was noted in various parts of the hippocampus, including the granular, polymorph and molecular layers of the dentate gyrus, the pyramidal cells, and the CA1–3 fields (Fig. 2A). Marked expression was also seen in cortical regions such as the piriform cortex, accessory basal amygdaloid nucleus (BMA) and posterolateral cortical amygdaloid nucleus (Fig. 2B). Moreover, *Slc16a14* mRNA was expressed in the arcuate nucleus and the dorsomedial and ventromedial hypothalamic nuclei (Fig. 2C).

### High degree of MCT14 expression in mouse brain

**Fig. 3:**
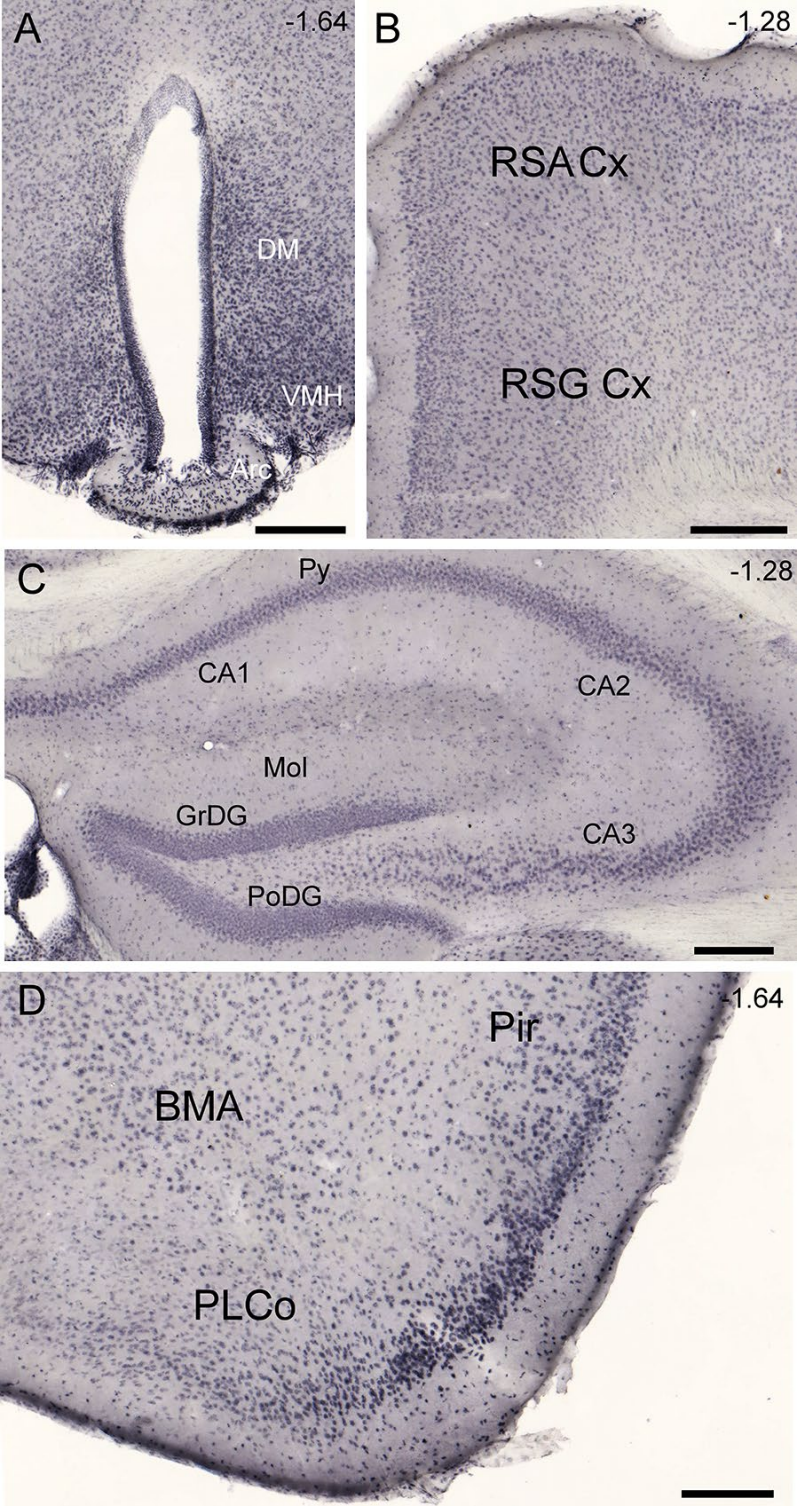
Widespread MCT14 expression in mouse brain. Close-up images of non-fluorescent IHC with DAB using the MCT14 antibody on 70-µm coronal mouse brain sections, to determine the expression pattern of MCT14. Expression was noted in the **A** arcuate nucleus (Arc) and ventromedial and dorsomedial hypothalamic nuclei (VMH and DM respectively), **B** retrosplenial agranular (RSA) cortex and retrosplenial granular (RSG) cortex, **C** granular, molecular and polymorph layers of the dentate gyrus (GrDG, Mol and PoDG respectively), CA1–3 fields and pyramidal cells of the hippocampus, **D** piriform cortex (Pir), postolateral cortical amygdaloid nucleus (PLCo) and accessory basal amygdaloid nucleus (BMA). All *scale bars* represent 200 µm. Bregma coordinates (*upper left corner* of each image) and abbreviations were determined according to Franklin and Paxinos [57]

Non-fluorescent IHC analysis of 70 µm coronal sections of mouse brain was performed with an anti-MCT14 antibody to map the MCT14 expression found in a variety of structures throughout the brain. Figure 3 depicts representative images of immunoreactivity in selected brain regions. Immunoreactivity was noted in the granular layer of the dentate gyrus, the CA1–3 fields, and in the pyramidal cells of the hippocampus (Fig. 3A). Staining was also seen in cortical regions such as the retrosplenial agranular cortex (Fig. 3B). The staining was widespread throughout the cortex and stretched across several distinct cortical regions such as the somatomotor cortex, the somatosensory cortex, and the occipital lobe. There was a wide regional distribution of staining in the hypothalamus, including the arcuate nucleus, and the ventromedial and dorsomedial hypothalamic nuclei (Fig. 3C).

### MCT14 is expressed in the soma of neurons and in epithelial cells

**Fig. 4:**
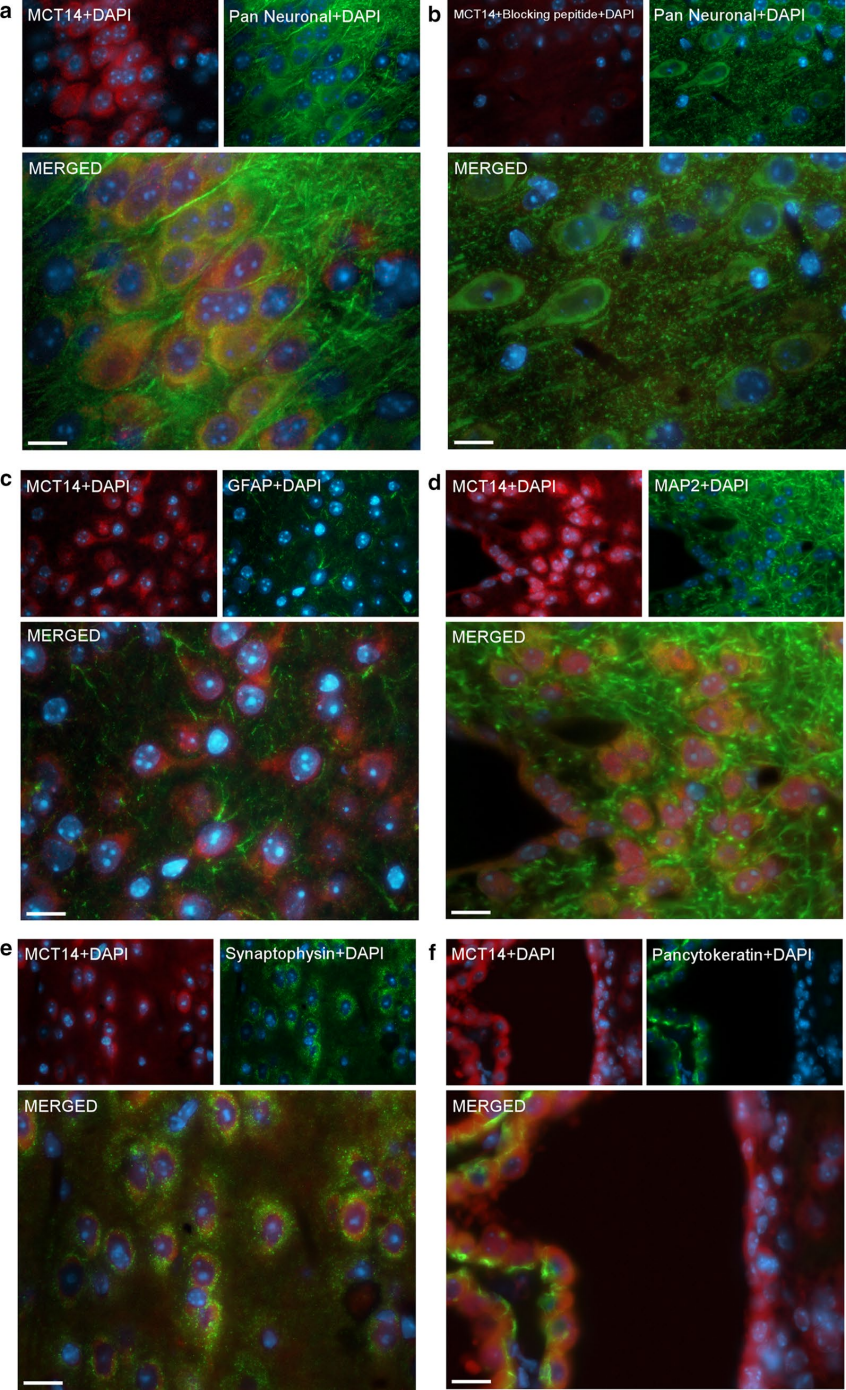
MCT14 is expressed in the soma of neurons and in epithelial cells. Fluorescent double IHC on paraffin sections from wild-type mouse brain with the MCT14 antibody and a panel of markers to identify the cell types expressing MCT14. The MCT14 antibody staining is seen in *red*, cell-specific markers are seen in *green*, and cell nuclei stained with DAPI are seen in *blue*. **a** Colocalization of MCT14 with the whole-neuron marker Pan neuronal cocktail aimed at soma, dendrites, axons and nuclei of neurons suggests that MCT14 is expressed in the neuronal soma in cortex. **b** Reduced MCT14 staining following pre-incubation of anti-MCT14 with a peptide corresponding to the antibody epitope, while Pan still stains the whole neuron. **c** No overlap between MCT14 and the astrocytic marker GFAP. **d** Overlap between MCT14 and neuronal marker MAP2 in the soma of neurons. **e** No colocalization of MCT14 and the pre-synaptic vesicle marker synaptophysin. **f** Colocalization of MCT14 and Pancytokeratin, an epithelial cell marker, indicates MCT14 expression in epithelial cells of the choroid plexus in the lateral ventricles. All images were taken at ×40 magnification on a Zeiss AxioImager, and all *scale bars* represent 10 µm

Fluorescent double IHC was performed on coronal 7-µm paraffin sections from wild-type mouse brains in order to identify the cellular localization of MCT14. The MCT14 antibody was used together with cell-specific antibodies for neurons, astrocytes, synapses and epithelial cells (Fig. 4). There was substantial overlap between MCT14 and Pan neuronal cocktail (Pan) [36], a whole-neuron marker for axons, dendrites, nuclei and soma of neurons. The staining was confined to the soma, and was absent in neuronal fibers (Fig. 4a). Double IHC with MCT14 and Pan was repeated, but with anti-MCT14 pre-incubated with the corresponding antigen. This blocks the antibody, preventing it from binding to the epitope in the brain sections. In Fig. 4b, MCT14-staining is absent or greatly reduced, while the Pan staining persists. This confirms the binding of anti-MCT14 to the specific epitope. The presence of MCT14 in the soma was further confirmed by the absence of colocalization with the dendritic marker, microtubuli-associated protein 2 (MAP2) [37] (Fig. 4c). Co-expression of MCT14 and the epithelial cell marker Pancytokeratin [38] in the epithelial cells surrounding the lateral ventricles suggests the presence of MCT14 in epithelial cells (Fig. 4d). The double IHC also revealed no overlap between MCT14 or for the marker for astrocytes [glial fibrillary acidic protein (GFAP) [39], Fig. 4e] or synaptophysin, a marker for pre-synaptic vesicles [40] (Fig. 4f).

### Intracellular and plasmalemmal expression of MCT14

**Fig. 5:**
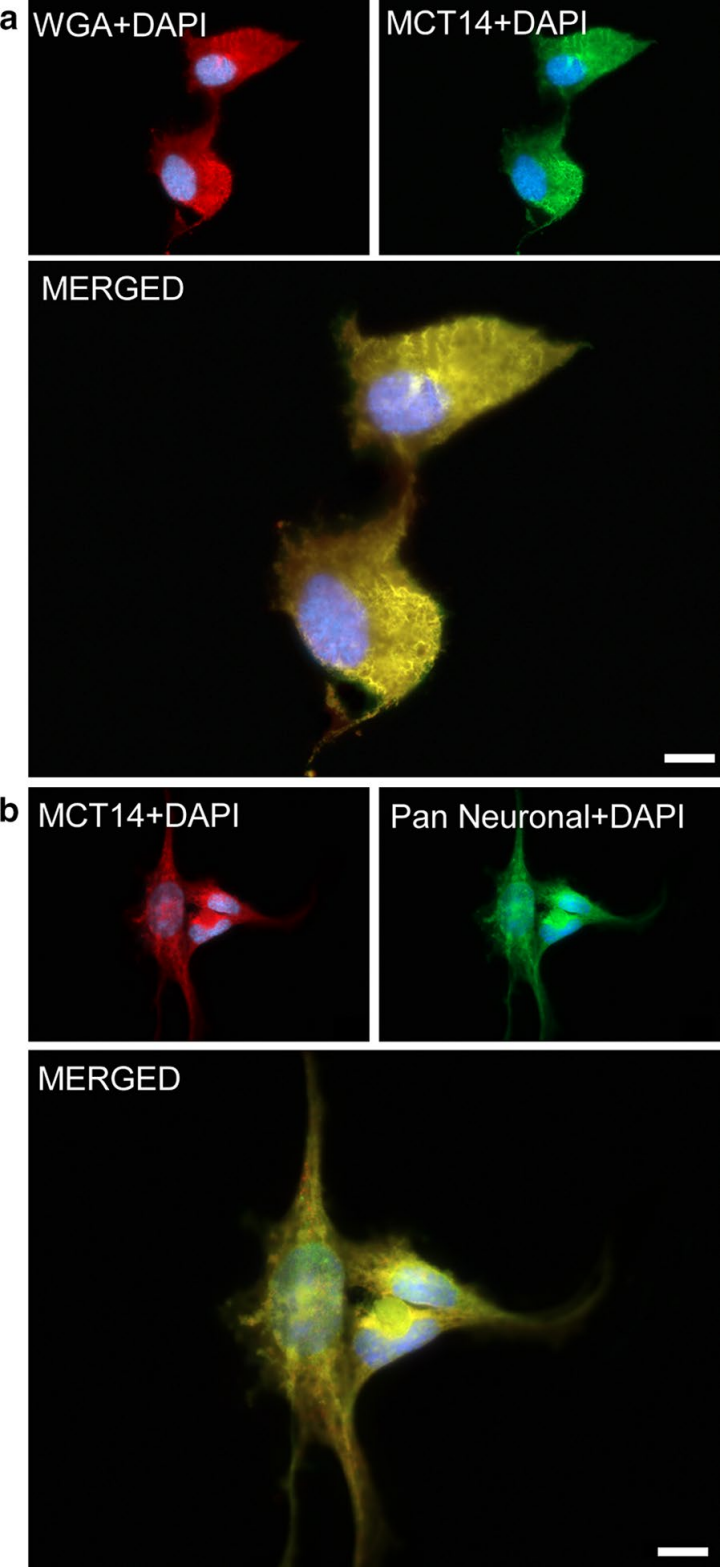
Intracellular and plasmalemmal localization of MCT14. Double ICC on fixed PC12 cells with anti-MCT14 and markers to further determine the localization of MCT14. **a** Overlap between MCT14 and WGA, a marker for plasma- and intra-cellular membranes. **b** Colocalization of MCT14 with whole neuron marker Pan neuronal cocktail indicates intracellular MCT14 expression as well as in the plasma membrane. All images were acquired at × 63 magnification on a Zeiss AxioImager. *Scale bars* represent 10 µm

The overlap between MCT14 and WGA (Fig. 5a), a marker for all membranes, indicates an intracellular expression of MCT14 as well as in the plasma membrane. This was verified with the double ICC with MCT14 and Pan, where the MCT14 and Pan staining overlapped in the PC12 cells (Fig. 5b).

### A majority of MCT14 positive neurons are non-GAD67

**Fig. 6:**
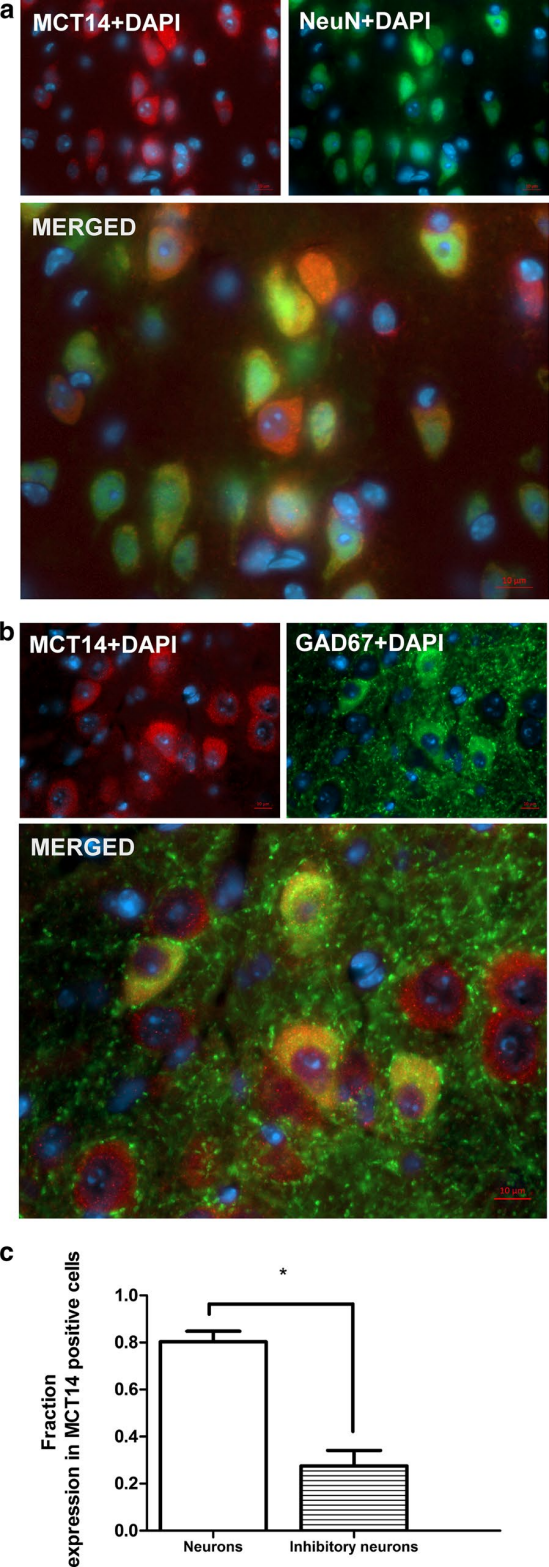
A majority of MCT14 positive neurons are non-GAD67. Double IHC on 7-µm paraffin sections with MCT14/NeuN and MCT14/GAD67 for determination of the type of neuron MCT14 is expressed in. MCT14 is seen in *red*, markers in *green*, and DAPI is seen in *blue*. **a** Colocalization of MCT14 with NeuN, a neuronal marker, in most MCT14-expressing cells. **b** Colocalization of MCT14 with neurons expressing GAD67, a marker for inhibitory neurons. **c** A total of six (n = 2) double IHC images with MCT14/NeunN and MCT14/GAD67 from retrosplenial cortex, hypothalamus and piriform cortex were collected and analyzed using a colocalization analysis pipeline from CellProfiler.org (Additional file 1: Figure S1). Fraction of MCT14-positive cells that also expressed respective marker was plotted. All images were acquired on a Zeiss AxioImager at ×20 magnification. Statistics were performed with Students’ t test, *p < 0.05

By using double IHC with neuronal markers together with the MCT14 antibody, we found that MCT14 was expressed in neurons. This led us to ask in which neuron type it was expressed. MCT14 displayed a high degree of colocalization with the neuronal marker NeuN (Fig. 6a). Furthermore, MCT14 also colocalized with a marker for inhibitory neurons, GAD67, albeit to a lesser extent (Fig. 6b). The degree of overlap between MCT14 and the markers was quantified using a specialized pipeline in automated cell segmentation software from CellProfiler.org (Additional file 1: Figure S1). Double IHC images of hypothalamus, cortex and piriform cortex from six sections (n = 2) for each region were analyzed with the colocalization pipeline. (For more details regarding the analysis modules included in the pipeline, please see Additional file 2.) The total fraction of MCT14-positive cells also positive for the neuronal marker was significantly higher than that of MCT14-positive cells that expressed the marker for inhibitory neurons (p = 0.0218) (Fig. 6c). Consequently, a dominant fraction of MCT14-positive neurons are non-GAD67.

### Verification of the MCT14 antibody

**Fig. 7:**
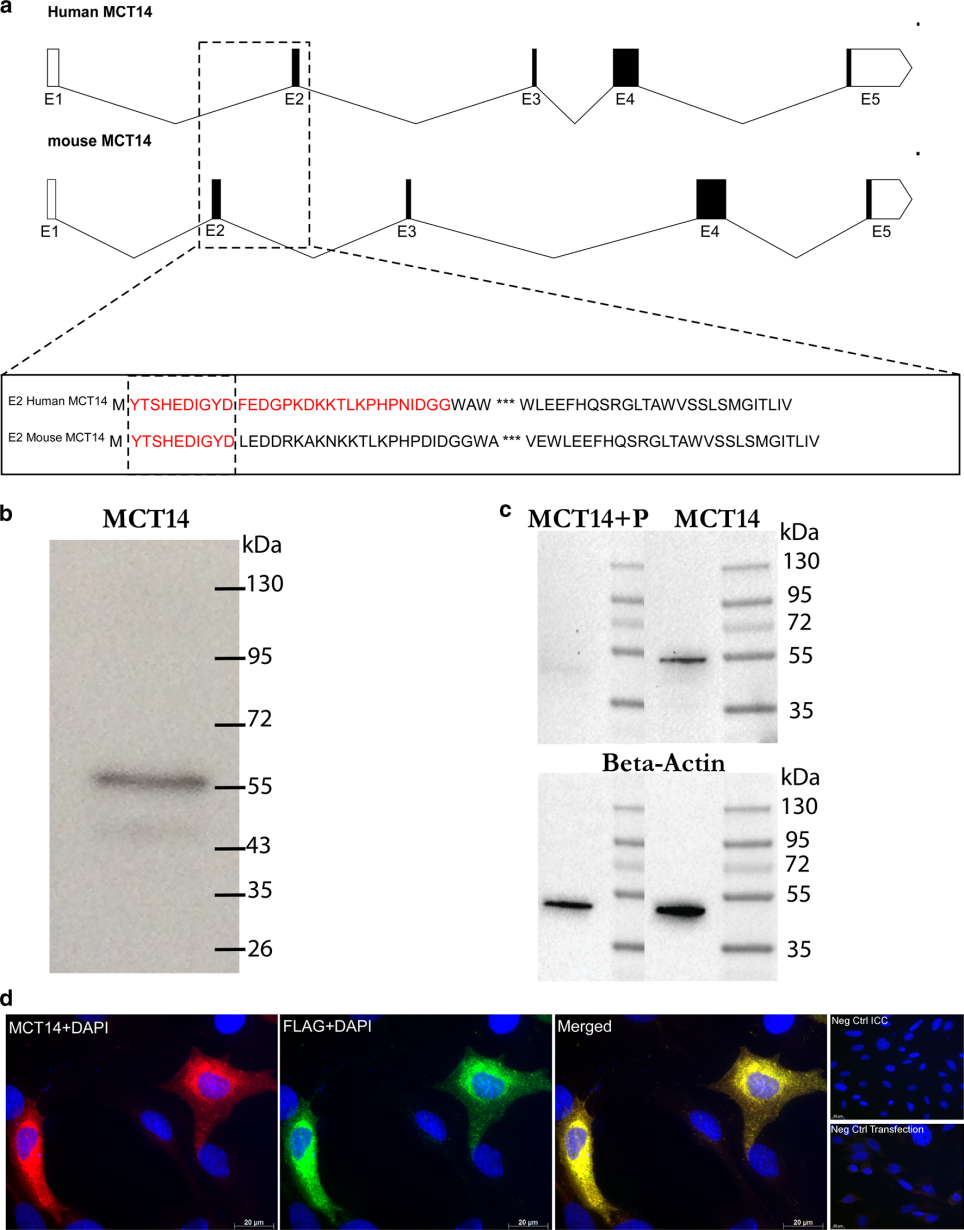
Validation of antibody specificity. The specificity of the antibody: **a** representation of the human *SLC16A14* and mouse *Slc16a14* and the epitopes for the MCT14 antibody in human and mouse. The epitope for the commercially available MCT14 antibody is found in the second exon (E2) in both the human and mouse MCT14 proteins. The *bottom most box* shows the peptide sequences encoded by E2 in human and mouse, respectively. The *underlined* sequence encoded by the human exon constitutes the epitope of the antibody (31 amino acids) and the *underlined* sequence encoded by the mouse E2 is the corresponding epitope in mouse (ten amino acids). Graphics were generated using the Exon–Intron Graphic Maker [58] and the *scale bars* (*upper right corners* of each gene) represent 100 bp. **b** A Western Blot on wild-type mouse brain homogenate was performed to analyze the specificity of the polyclonal anti-MCT14 antibody. It generated a strong band at ~56 kDa corresponding to the mouse MCT14 protein (NP_082197) and a weaker band at ~45 kDa corresponding to the 48 kDa mouse isoform (EDL02188.1). **c** A peptide competition with anti-MCT14 and peptide corresponding to the epitope recognized by the antibody. The *right lane* shows a Western Blot of anti-MCT14 on wild-type mouse brain homogenate, resulting in a band at ~56 kDa. The *left lane* shows anti-MCT14 neutralized with an excess of peptide, resulting in a very faint band with the same size. *Bottom blot* shows the loading control, β-actin, with expected bands around 42 kDa. Densitometric analysis of MCT14 bands normalized against the β-actin control showed a 40 % reduction of band intensity when blocked antibody was blotted. **d** Double IHC on immortalized PC12 cells transfected with FLAG-tagged plasmids containing the full *Slc16a14* mRNA sequence with the MCT14 antibody and FLAG antibody revealed colocalization of MCT14 and the FLAG. MCT14 staining is red, the FLAG marker is green and cell nuclei are stained with DAPI. Negative control for the immunocytochemistry (no primary antibodies) and for the transfection (no cDNA during transfection) can be seen in the *upper* and *lower right corners*, respectively. All images were acquired with ×40 magnification

The commercially available anti-MCT14 polyclonal antibody used in the histological analysis was raised against the human MCT14 protein; the epitope for the antibody is located in the second exon in both the human and mouse proteins (Fig. 7a) The epitope for the human MCT14 protein is YTSHEDIGYDFEDGPKDKKTLKPHPNIDGG and the corresponding sequence in mouse is YTSHEDIGYD, i.e. the first ten amino acids. BLAST analysis [41, 42] confirmed a high degree of homology between the human MCT14 protein and the two mouse isoforms (87 and 92 % identity of query coverage) and the two mouse isoforms (100 % identity of the query coverage). To verify the antibody specificity, a Western Blot analysis was performed using the MCT14 antibody on homogenized wild-type mouse brain. Analysis of the obtained blot revealed two bands at ~56 and ~45 kDa (Fig. 7b), corresponding to the theoretical molecular mass of the two MCT14 isoforms in mouse, NCBI RefSeq NP_082197 (512 amino acids, 56.4 kDa) and GenBank ID EDL02188.1 (436 amino acids, 48 kDa). The theoretical molecular mass of the latter was calculated using the peptide sequence in Protein Calculator v3.4 [43, 44]. The results from Western Blot are in line with the sequence analysis revealing that MCTs are not likely to be glycosylated, and that bands on the Western Blot cannot be shifted by deglycosylation [44, 45]. Western Blot was also performed on anti-MCT14 that had been neutralized with a peptide corresponding to the epitope recognized by the antibody (Fig. 7b). By binding the peptide, the antibody is blocked from binding the epitope present on the blot. The blotting of peptide-bound anti-MCT14 resulted in a very faint band with the predicted molecular size, compared to the control sample with anti-MCT14 only. Band intensities were quantified and normalized with β-actin, and showed a 40 % reduction of signal in the peptide-blocked blot. Further verification of the antibody was attained by simultaneous immunodetection of MCT14 and the FLAG sequence on PC12 cells transfected with a pcDNA3.1 + DYK plasmid containing the full *Slc16a14* mRNA sequence and FLAG tag. Double ICC with the MCT14 and the FLAG antibody (Fig. 7b) reveals a colocalization of MCT14 and the immunogen FLAG epitope DYKDDDK.

### Moderate to high Slc16a14 mRNA expression in CNS, testis, uterus, liver and kidney

**Fig. 8:**
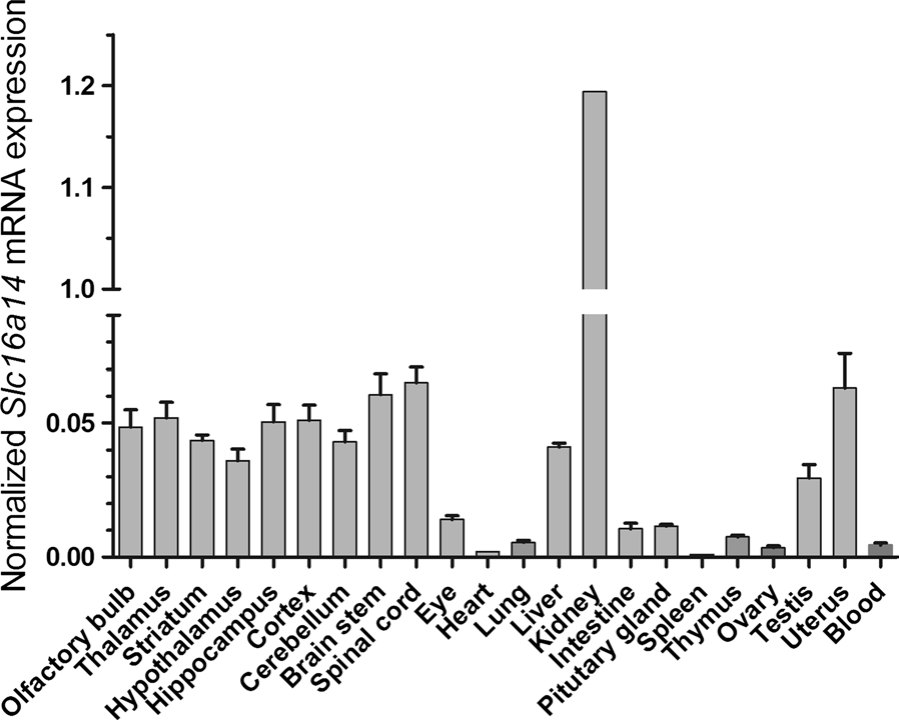
High *Slc16a14* mRNA levels in kidney and moderate levels in the CNS, testis, uterus and liver. Quantitative real-time PCR on wild-type mouse brain regions and peripheral tissues presented as mean ± standard deviation (SD). High *Slc16a14* mRNA expression in wild-type mouse kidney and moderate levels in the CNS, testis, uterus and liver, while the levels in other tissues were low

Quantitative RT-PCR was performed on a panel of sub-dissected mouse brain regions and peripheral tissues (Fig. 8) to obtain an overview of the *Slc16a14* mRNA expression. RT-PCR was used to measure normalized mRNA expression levels (± SD) (Fig. 7a) and shows a varied expression of *Slc16a14* throughout the samples. Moderate levels of *Slc16a14* mRNA were expressed in most CNS regions as well as in testis, uterus and liver. The highest *Slc16a14* mRNA levels were measured in the kidneys, approximately 20-fold higher than the levels in CNS.

## Discussion

Only 6 of the 14 members of the MCT family have been studied extensively: MCT1–4, MCT8, and MCT10. The remaining transporters have yet to be characterized in detail. Here we aimed to elucidate the tissue distribution of MCT14, determine its cellular localization, and study its evolutionary relationship to other members of the monocarboxylate transporter family.

We performed a phylogenetic analysis revealing a close relationship between *Slc16a14* and *Slc16a2*, *Slc16a9*, and *Slc16a10*. QRT-PCR on mouse brain and a panel of peripheral mouse tissues revealed moderate expression levels of *Slc16a14* throughout the CNS and high levels in the kidney, approximately 20-fold higher than in the CNS. To obtain a more detailed mRNA mapping in the CNS, the expression pattern of *Slc16a14* was determined through ISH using an antisense probe. *Slc16a14* exhibited widespread distribution in a variety of structures in the brain, including the hippocampus, hypothalamus as well as cortical and subcortical regions. This was in line with *Slc16a14* expression data from the Allen Brain Atlas [46, 47] and was similar to the expression data available for the most closely related transporters in the MCT family. The *Slc16a9* expression data are only found in the Allen Brain Atlas, whereas *Slc16a2* and *Slc16a10* have been studied to a greater extent. The expression of *Slc16a2* in brain has been established by several independent studies [32, 47–49], which locate it in the hypothalamus, hippocampal and cortical areas, as well as amygdala and choroid plexus. *Slc16a10* is closely related to *Slc16a2*, but seems to be expressed to a lesser extent in the brain, mainly in hypothalamus and hippocampus [50], and the expression seems to increase with age.

For the histological analysis of MCT14, a commercially available rabbit polyclonal antibody was used. We performed extensive validation of the antibody using Western Blot, peptide competition with both Western Blot and IHC. We also performed a double IHC on PC12 cells transfected with a *Slc16a14*-containing FLAG-tagged plasmid and subsequent double IHC with anti-MCT14 and anti-FLAG. The staining of the MCT14 and FLAG overlapped. On the basis of these data, we concluded that the MCT14-antibody is specific.

The distribution of MCT14 matches that of the mRNA, as determined by non-fluorescent IHC. MCT14 is expressed in various parts of the brain, including the hypothalamus, hippocampus and cortex. The close evolutionary relationship between MCT14 and MCT8, 9, and 10 is in line with the overlap in tissue distribution, in that they are all highly expressed in mouse brain and kidneys [14]. Our phylogenetic analysis showing the close relationship between *Slc16a14* and *Slc16a2*, *Slc16a9* and *Slc16a10* is further supported by studies indicating that carnitine, transported by MCT9, is capable of negating the effect of thyroid hormones, transported by MCT8 and MCT10 [51, 52]. For carnitine to be a possible inhibitor, similarities in the binding site and the protein fold are necessary, and these features are primarily determined by sequence.

To further investigate the cellular localization of MCT14, fluorescent IHC was used with cell-specific markers. MCT14 was expressed in the soma of neurons as well as epithelial cells, but absent in astrocytes and in synapses. Colocalization analysis revealed MCT14 expression in both GAD67 and non-GAD67 expressing neurons. Given that the majority of non-inhibitory neurons in the brain are excitatory, we conclude that MCT14 is expressed in both excitatory and inhibitory neurons. The staining was confined to the soma and absent in projections, indicating a plasmalemmal and intracellular localization of MCT14. The cell-specific expression of MCT14 in epithelial cells and neurons is also similar to that of MCT8, which is expressed in all cell types in CNS except microglia, with the exception of MCT8 expression in astrocytes [53] where MCT14 is absent; MCT10 on the other hand seems to be specifically expressed in white matter regions in the brain, indicating MCT10 enrichment in oligodendrocytes [50].

The physiological roles of the characterized MCTs differ, depending on their substrate profile and their differential expression in various tissues, as evidenced by the expression of distinct MCT isoforms. The substrate profile of MCT9 is still unknown, although it has been shown to transport carnitine [19, 20]. The key residues that seem to be involved in proton translocation in the proton-linked transport of MCT1–4 required to transport monocarboxylates are not conserved in MCT9, MCT8, MCT10 or MCT14 [13, 14]. The transport of thyroid hormones and aromatic amino acids across membranes by MCT8 and MCT10 is indeed proton-independent [15–17]. Taking all of this into account, it is likely that MCT14 neither transports monocarboxylates nor transports other possible substrates in a proton-dependent manner. Given its wide distribution pattern with the highest mRNA levels in the kidney and CNS, the substrate profile could include hormones involved in renal function, amino acids and/or ions. For the coordination of growth and development, amino acid transporters are important in the delivery of amino acids to the brain [54], nutrient sensing [55, 56] and subsequent regulation of food intake. Sequence similarities with amino acid transporters within the same family, the widespread tissue distribution, and its localization on the soma of epithelial cells and neurons contribute to the hypothesis that MCT14 is a transporter of amino acids.

## Conclusions

To summarize, we have presented a phylogenetic analysis revealing that *Slc16a14* is closely related to *Slc16a2*, *Slc16a9* and *Slc16a10*. We have provided a detailed expression analysis of *Slc16a14* expression, finding widespread expression in the mouse brain. Using a verified antibody, we performed a detailed immunohistochemical analysis of MCT14, establishing its expression in neurons and epithelial cells. QRT-PCR on a panel of mouse tissues revealed high *Slc16a14* expression in the kidney and moderate levels in the CNS. Further studies are needed to determine the endogenous substrates for MCT14 and to determine the mode of transport to establish the role of MCT14 in physiological and pathological processes.

## Additional files

10.1186/s12868-016-0274-7 An analysis pipeline from CellProfiler to measure the colocalization of MCT14 with NeuN and GAD67. The images from double immunohistochemistry were split into the separate channels but analyzed in the same run. The first four modules are input modules, and the remaining ones are for analysis. First, images were aligned and corrected for uneven illumination (modules 5-7). MeasureCorrelation (module 8) gave a general sense of the correlation of the stains in the respective images. For object-based correlation of staining, criteria for shape, size and threshold were set in IdentifyPrimaryObjects (module 9). The segmented objects were then related to each other (module 10). To find colocalized objects that shared the same center, objects were shrunk and then expanded to two pixels (modules 11 and 12), after which these points were related to each other (module 13). The colocalized objects were classified and filtered for un-colocalized noise. Statistics were exported to an Excel spreadsheet for further analysis (modules 14-18).
10.1186/s12868-016-0274-7 Experimental procedures.
10.1186/s12868-016-0274-7 Clone map of the expression vector containing the FLAG marker and the full *Slc16a14* sequence. Map drawn with Snapgene (www.snapgene.com).
