# Supplementary figures and images for: Histological characterization of orphan transporter MCT14 (SLC16A14) shows abundant expression in mouse CNS and kidney
Source: BMC Neurosci. 2016 Jul 1;17:43. doi: 10.1186/s12868-016-0274-7 (PMC4929735; doi:10.1186/s12868-016-0274-7)

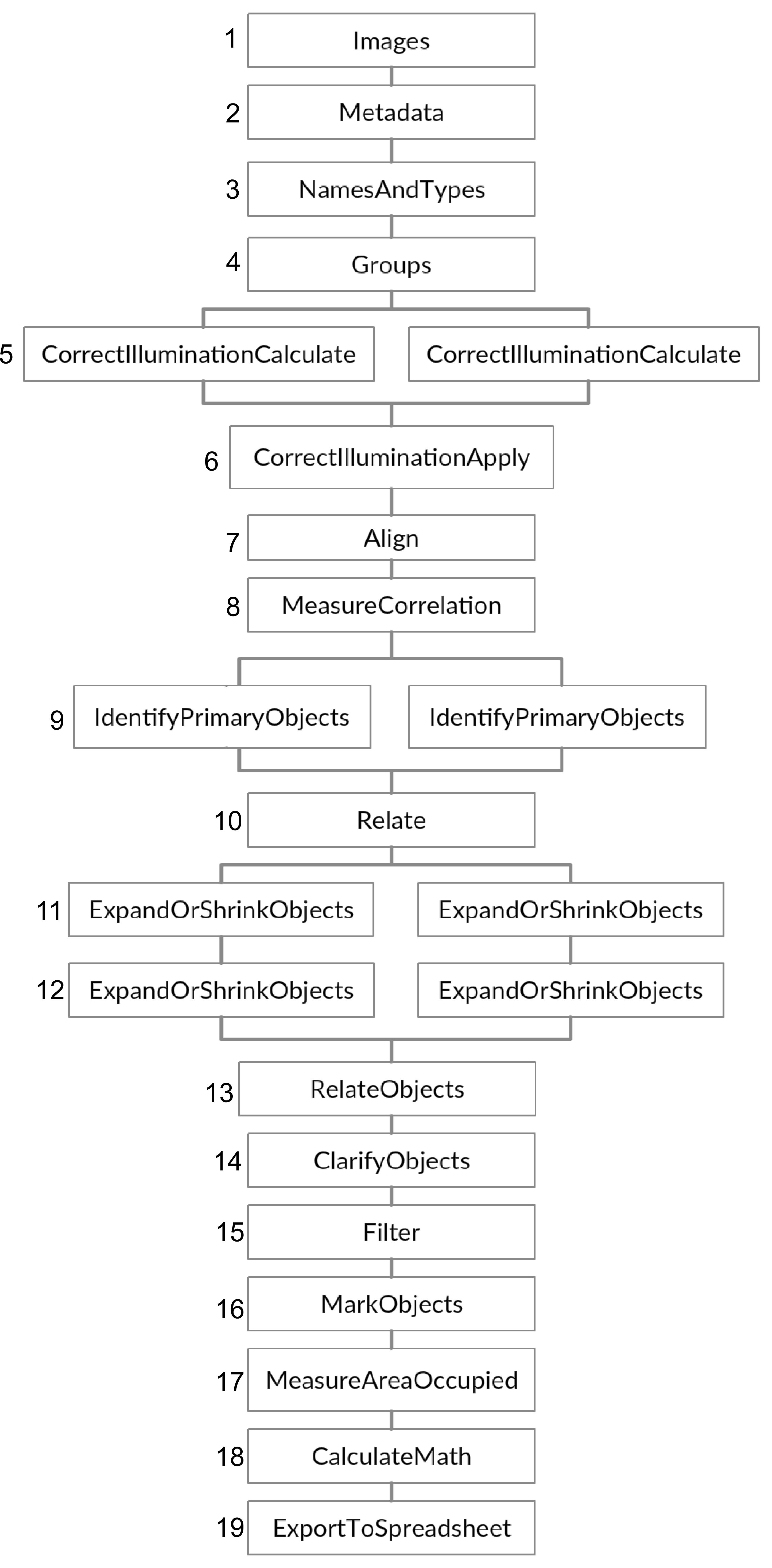

Supplement: Supplementary file 1 — 10.1186/s12868-016-0274-7 An analysis pipeline from CellProfiler to measure the colocalization of MCT14 with NeuN and GAD67. The images from double immunohistochemistry were split into the separate channels but analyzed in the same run. The first four modules are input modules, and the remaining ones are for analysis. First, images were aligned and corrected for uneven illumination (modules 5-7). MeasureCorrelation (module 8) gave a general sense of the correlation of the stains in the respective images. For object-based correlation of staining, criteria for shape, size and threshold were set in IdentifyPrimaryObjects (module 9). The segmented objects were then related to each other (module 10). To find colocalized objects that shared the same center, objects were shrunk and then expanded to two pixels (modules 11 and 12), after which these points were related to each other (module 13). The colocalized objects were classified and filtered for un-colocalized noise. Statistics were exported to an Excel spreadsheet for further analysis (modules 14-18). [file 12868_2016_274_MOESM1_ESM.tif]

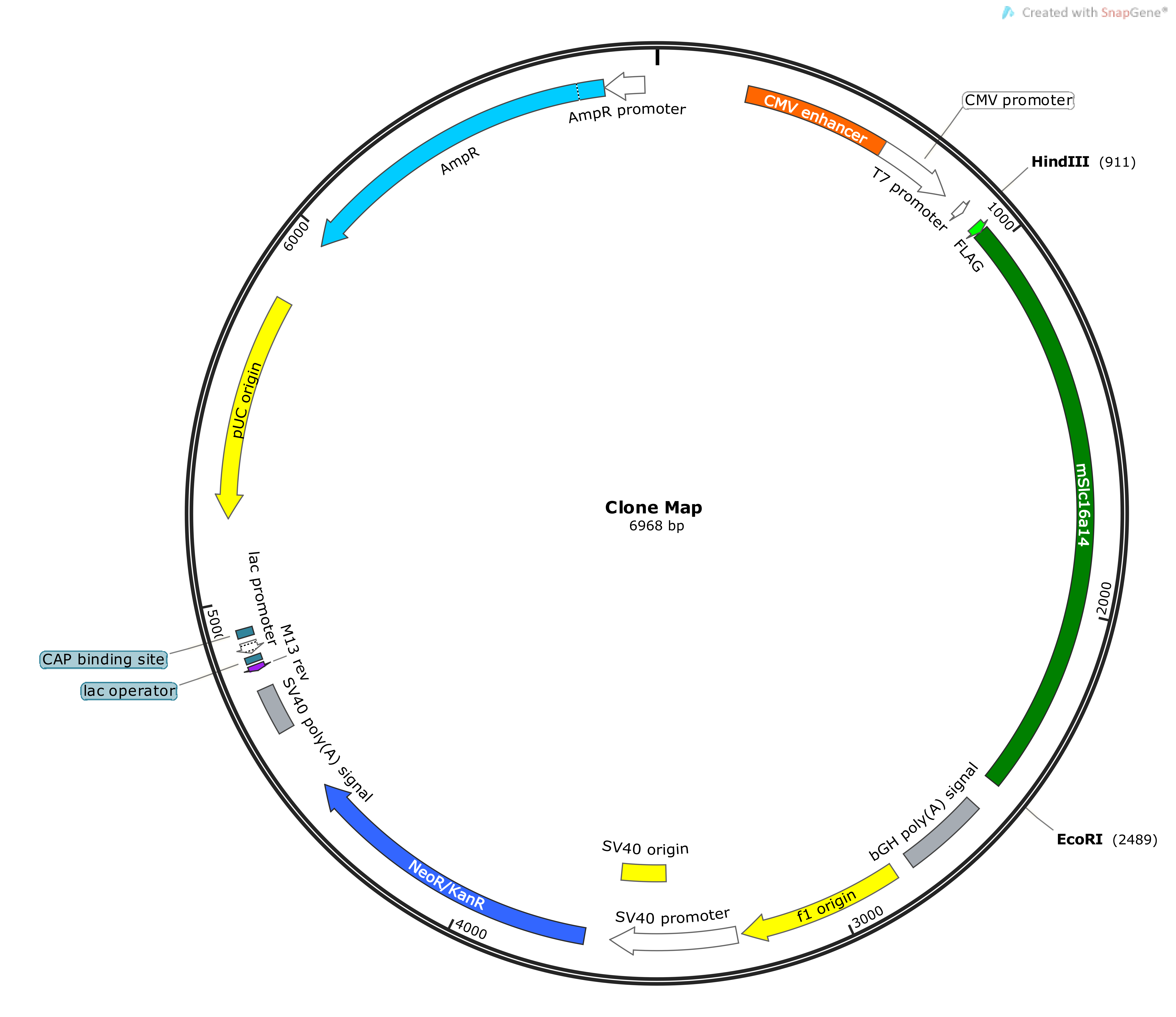

Supplement: Supplementary file 3 — 10.1186/s12868-016-0274-7 Clone map of the expression vector containing the FLAG marker and the full Slc16a14 sequence. Map drawn with Snapgene (www.snapgene.com). [file 12868_2016_274_MOESM3_ESM.tiff]
