# Supplementary material for: Histological characterization of orphan transporter MCT14 (SLC16A14) shows abundant expression in mouse CNS and kidney
Source: BMC Neurosci. 2016 Jul 1;17:43. doi: 10.1186/s12868-016-0274-7 (PMC4929735; doi:10.1186/s12868-016-0274-7)
Supplement: Supplementary file 2 — 10.1186/s12868-016-0274-7 Experimental procedures. [file 12868_2016_274_MOESM2_ESM.docx]

# Supplementary information

# Methods

Colocalization analysis
Colocalization of MCT14/NeuN and MCT14/GAD67 in double immunohistochemistry images were analyzed using an object-based colocalization analysis pipeline (Suppl. Fig.1). The colocalization was measured with an object-based method. The two images were aligned and corrected for uneven illumination before being separately segmented into appropriate objects using shape, size and threshold levels as segmentation criteria. These settings were optimized for each image set. Thereafter, objects were deemed to be related to each other if they shared the same center localization, and this was achieved by shrinking the objects to a point and then expanding them by two pixels. Lastly, colocalized objects were classified and filtered, after which statistics and colocalization counts were exported to an Excel spreadsheet.
